# Motor control strategies during double leg squat following anterior cruciate ligament rupture and reconstruction: an observational study

**DOI:** 10.1186/1743-0003-11-19

**Published:** 2014-02-28

**Authors:** Paulien E Roos, Kate Button, Robert W M van Deursen

**Affiliations:** 1 School of Healthcare Sciences, Cardiff University, Heath Park, Cardiff CF14 4XN, UK; 2 Arthritis Research UK Biomechanics and Bioengineering Centre, Cardiff University, Cardiff, UK; 3 Cardiff and Vale University Health Board, Cardiff, UK

**Keywords:** Anterior cruciate ligament, Biomechanics, Rehabilitation, Knee, Squat

## Abstract

**Background:**

Anterior cruciate ligament (ACL) injured individuals often show asymmetries between the injured and non-injured leg. A better understanding of the underlying motor control could help to improve rehabilitation. Double leg squat exercises allow for compensation strategies. This study therefore investigated motor control strategies during a double leg squat with the aim to investigate if individuals with ACL rupture (ACLD), ACL reconstruction (ACLR) and healthy control subjects (CONT) used different strategies.

**Methods:**

20 ACLD and 21 ACLR were compared to 21 CONT subjects. Participants performed eight continuous double leg squats to their maximum depth, while kinematic and kinetic data were collected. Outcome measures were calculated to quantify the behavior of the injured and non-injured legs and the asymmetry between these legs.

**Results:**

Squat depth was significantly reduced in ACLR and ACLD compared to CONT (p < 0.05; 106 ± 17°; 105 ± 21°; 113 ± 21°). Peak knee extensor moments (*M*_*kn(mx)*_) were significantly reduced in ACLR and ACLD compared to CONT in the injured leg only (p < 0.05; 0.045 ± 0.015; 0.046 ± 0.016; 0.059 ± 0.022 body weight.height respectively). There was no significant correlation between symmetry of the support moment (*SYM*_*Msup*_) and of the % support moment by the knee (*SYM%sup*_*kn*_) in CONT (R^2^ = -0.07). Data distribution average indicated good symmetry. ACLR showed a significant correlation between *SYM*_*Msup*_ and *SYM%sup*_*kn*_ (R^2^ = 0.561) when two participants who did not recover as well were excluded. ACLR *controlled knee moment* magnitude using two strategies; 1) transfer of support moment to non-injured leg; 2) transfer of support moment from knee to ankle and/or hip of injured leg. These were combined in different proportions, but with the same effect on the knee moment. ACLD showed no significant correlation between *SYM*_*Msup*_ and *SYM%sup*_*kn*_ (R^2^ = 0.015). Data distribution average indicated reduced symmetry. ACLD therefore used an *avoidance strategy*: reducing squat depth and subsequently the support moment in the injured leg and the knee contribution.

**Conclusions:**

ACLD and ACLR individuals used different squatting strategies compared to controls, with ACLR using controlled and ACLD using avoidance behavior regarding knee loading. This has major implications for rehabilitation as these kinetic strategies cannot be observed, but result in the injured leg not being exercised as intended.

## Background

Anterior Cruciate Ligament (ACL) injury often results in failure to return to pre-injury activity levels [1-4]. Approximately 42-65 % of ACL injured individuals who had reconstructive surgery [1-3] and 18-50 % of those who were treated conservatively [2,4] do not return to their pre-injury levels. ACL injury also leads to an increased predisposition of early-onset osteoarthritis [5], affecting 50 % of ACL deficient individuals (ACLD) [6] and 33-39 % of individuals with ACL reconstruction (ACLR) [7,8]. The mechanisms from injury to early onset osteoarthritis are unknown, although altered loading of the knee has been proposed as one important potential mechanism [9-11]. Better insight into altered kinematics and kinetics during functional activities is therefore needed. Numerous studies have highlighted that there are asymmetries in kinematics or kinetics between the injured and non-injured leg in ACL patients [12-19]. Asymmetries persisted even six months to two years post-surgery [13,16,17,19]. Such asymmetries could result in altered loading of the knee joint. Although asymmetric behavior has been reported, insight into the motor control strategies behind the asymmetries is limited. A better understanding of the motor control of these asymmetries should help to improve rehabilitation of ACL injured patients.

Double leg squat exercises are used in early rehabilitation of ACL injured individuals to strengthen quadriceps and hamstring muscles and to inform treatment selection [20-22]. Asymmetric behavior will however limit the effectiveness of this exercise which allows for compensation strategies because it involves multiple joints and bilateral leg support. It appears that ACLR patients adopt such asymmetric strategies during this exercise [14], with a reduced peak knee extensor moment and a trend to an increased peak hip extensor moment in the injured compared to the non-injured leg [14]. The loading response has also been found to be asymmetric in ACLR patients and reduced in the injured compared to the non-injured leg [23]. This suggests a compensation mechanism of reduced effort of the knee and increased effort of the hip in the injured leg. The sample size in Salem et al. [14] was however too small to demonstrate a significantly increased effort of the hip and to investigate the motor control strategies underlying these compensations. The motor control strategies behind the asymmetries during a double leg squat in ACL injured individuals therefore remain not well understood. A better understanding of such strategies is essential to be able to address them in rehabilitation. Furthermore, no studies have investigated double leg squatting strategies in ACLD patients.

This study therefore investigated motor control strategies during a double leg squat with the aim to investigate if individuals with ACL rupture (ACLD), ACL reconstruction (ACLR) and healthy control subjects (CONT) used different strategies. It was hypothesized that both ACLR and ACLD would use compensation strategies during the double leg squat with a decreased effort of the knee and an increased effort of the hip in the injured compared to the non-injured leg and that these strategies would differ between ACLR and ACLD.

## Methods

### Participants

20 ACLD (height: 1.80 ± 0.08 m, mass: 82.9 ± 12.5 kg, age: 29 ± 6 years, gender: 3 female, 17 male) and 21 ACLR (height: 1.73 ± 0.07 m, mass: 80.1 ± 9.5 kg, age: 29 ± 9 years, gender: 5 female, 16 male) were compared to 21 CONT subjects (height: 1.75 ± 0.13 m, mass: 77.6 ± 19.6 kg, age: 27 ± 8 years, gender: 9 female, 12 male). Participant numbers were based on power calculations; using means and standard deviations from Salem et al. [14] we calculated an effect size [24] of 0.84, when using an alpha of 0.05 and power of 0.8 this resulted in 21 participants in each group.

There was no significant difference in characteristics (p = 0.093, p = 0.506 and p = 0.540 for height, mass and age respectively) between the participant groups. There was no significant difference in time since injury between ACLD and ACLR groups (ACLR: 24 ± 17 months; ACLD: 19 ± 52 months; p = 0.693; Table 1). All ACLR participants had a single bundle four strand gracilis-semitendinosus tendon graft reconstruction and were at least 6 months post-surgery. All participants provided informed consent to take part in this study and ethical approval was obtained from South East Wales Local Research Ethics Committee. Inclusion criteria were that ACLR and ACLD patients were aged between 18 and 65 years; had an ACL rupture that may or may not be accompanied with a meniscal tear or collateral ligament sprain; had a primary ACL reconstruction (for ACLR group only); had finished their rehabilitation; had no other pathology which affects their movement; had no previous knee surgery and were able to provide informed consent independently. Inclusion criteria for CONT were that subjects were aged between 18 and 65 years; had no knee injury, knee surgery or other pathology which affects their movement and were able to provide informed consent independently.

**Table 1:** Participant strength and questionnaire data

	**T**_**inj **_**(mths)**	**T**_**surg **_**(mths)**	***S***_***KnExt ***_**(BW.h)**	***S***_***KnFlex ***_**(BW.h)**	**CSAS**	**TSK**	**IKDC**
CONT	-	-	0.105 ± 0.026	0.061 ± 0.014	87 ± 17	-	-
ACLR	24 ± 17	13 ± 9	0.096 ± 0.039	0.057 ± 0.017	82 ± 16	32.7 ± 4.9	83 ± 10
ACLD	19 ± 52	-	0.083 ± 0.033*	0.054 ± 0.016*	72 ± 19*	41.0 ± 5.1&	61 ± 12&

Mean time since injury (T_inj_), time since surgery (T_surg_), knee extensor (*S*_*KnExt*_ and *S*_*KnFlex*_) and knee flexor strength, Cincinnati Knee Rating System Sports Activity Scale (CSAS), Tampa Scale of Kinesiophobia (TSK) and International Knee Documentation Subjective Knee questionnaire (IKDC) scores with standard deviations. A * indicates a significant difference (p < 0.05) from CONT, and a & significant difference from ACLR (p < 0.05).

### Patient rated questionnaires and strength measurement

For ACLD and ACLR subjects knee-specific symptoms, function and sports activity was scored as a single measure using the International Knee Documentation Subjective Knee (*IKDC*) questionnaire [25]. Fear of re-injury was scored for ACLD and ACLR subjects using the Tampa Scale of Kinesiophobia (*TSK*) [26] with adaptations for knee injury specific fear of re-injury as in [27]. Activity level was scored (general for CONT and post-injury for ACLD and ACLR) using item 3 (Sports Activity Scale (*CSAS*)) from the Cincinnati Knee Rating System [28]. Isokinetic knee extensor (*S*_*KnExt*_) and flexor (*S*_*KnFlex*_) strength were measured at 60°/s on a Biodex System 4 PRO dynamometer (Biodex Medical Systems Inc, USA). This was measured through the full range of motion and over five repetitions without any rest in between. The subjects were sitting upright with the hip at approximately 90° flexion and the thigh fixed to the dynamometer chair. Although this was measured on both legs, data are presented for the injured (ACLR and ACLD) and the dominant stance leg (CONT) only.

### Data collection and processing

Participants were asked to perform eight continuous double leg squats to their maximum depth. The feet were placed each on a separate force platform. No further instructions were given on foot placement, upper limb position or squatting technique in order to observe the participants’ normal behavior. Prior to this a static anatomical calibration trial was collected. Kinematic data were collected at 250 Hz using an eight camera VICON MX motion analysis system (Oxford Metrics Group Ltd., UK). Reflective markers were placed using the ‘Plug-in-Gait’ full body marker set. Two additional markers were placed on the left and right lateral sides of the iliac crest (LILC and RILC). Ground reaction force data were collected using two Kistler force plates (Kistler Instruments Ltd., Switzerland) at 1,000 Hz. Ground reaction force data were filtered with a fourth order Butterworth filter and a low pass cut off frequency of 12 Hz, and marker data with a fourth order Butterworth filter and a low pass cut off frequency of 20 Hz. In most trials the markers on the left and right anterior superior iliac crests (LASI and RASI) were occluded during the deepest point of the squat; these gaps were filled using a custom written program in Vicon BodyBuilder for Biomechanics (version 1.2, Oxford Metrics Group Ltd., UK) and the data of the LILC and RILC markers. This program reconstructed any of the six marker positions missing in the dynamic trial by exploiting the redundancy and using the exact position of any missing markers relative to a pelvis reference frame defined in the static calibration trial. The knee axes were aligned using the anatomical calibration trial.

### Data analysis

Inverse kinematics and dynamics calculations were performed within Vicon Nexus software (version 1.6.1) and data were further processed and analyzed in Matlab R2010b (The Mathworks Inc., USA). Anthropometric measurements were recorded (height, mass, leg length, knee width and ankle width and used for the inverse dynamic calculations. Output parameters (after Winter [29]) were calculated in Matlab and were as follows, with variables with the subscript ending in I relating to the injured leg and ending in N to the non-injured leg (in CONT N and I were randomly taken as the left or right leg): *α*_*kn(mx)*_: peak knee flexion angles; *M*_*kn(mx)*_: peak knee extensor moments; *M*_*sup*_: support moment (sum of the ankle plantar flexor and knee and hip extensor moments) at *M*_*kn(mx)*_; *Msup*_*ank*_ the percentage of *Msup* produced by the ankle; *Msup*_*kn*_ the percentage of *Msup* produced by the knee; *Msup*_*hip*_ the percentage of *Msup* produced by the hip; *SYMα*_*kn(mx)*_: symmetry of the peak knee flexion angles between the injured and non-injured legs; *SYM*_*Msup*_: symmetry of the support moment between the injured and non-injured legs; *SYM%sup*_*kn*_: symmetry of the % support moment of the knee between the injured and non-injured legs; *SYM%sup*_*ankle*_: symmetry of the % support moment of the ankle between the injured and non-injured legs; *SYM%sup*_*hip*_: symmetry of the % support moment of the hip between the injured and non-injured legs. Symmetry was calculated as follows [29]:

$$\text{Symmetry}=\frac{2*\text{Injured}}{\text{Injured}+\text{Non}-\text{injured}}*100$$

### Statistical analysis

After checking for normal distribution, a one-way ANOVA was used for the normal distributed kinematic and kinetic output variables and a Kruskal-Wallis test for the not normal distributed symmetry measures to investigate differences between ACLR and CONT and between ACLD and CONT. To investigate trends between the symmetry measures simple linear regression analysis was used between *SYM%sup*_*ankle*_ and *SYM%sup*_*kn*_, and between *SYM%sup*_*hip*_ and *SYM%sup*_*kn*_. An alpha level of p < 0.05 was used to evaluate statistically significant between-groups difference.

## Results and discussion

### Participant characteristics

ACLD had significantly lower knee flexor (*S*_*KnFlex*_) and extensor strength (*S*_*KnExt*_) than CONT (p < 0.001), while strength of ACLR was not significantly different from CONT (p = 0.068; p = 0.057) when strength was normalized to body weight and height (Table 1). ACLD had a significantly lower activity level than CONT (p = 0.020; *CSAS*; CONT: 87 ± 17; ACLR: 82 ± 16; ACLD: 72 ± 19), higher fear of re-injury (p = <0.001; *TSK*; ACLR: 32.7 ± 4.9; ACLD: 41.0 ± 5.1) and lower knee function (p = <0.001; *IKDC*; ACLR: 83 ± 10; ACLD: 61 ± 12) than ACLR (Table 1). ACLD on average had fear of re-injury while ACLR did not, as a *TSK* score above 37 has been associated with the presence of fear of re-injury. CONT had a larger proportion of females than ALCD and ACLR. Data were normalized to height and weight to take away the main gender effects. Some gender differences have been reported for limb symmetry, with females being more asymmetric [30]. The smaller proportion of females in the ACLR and ACLD group in our study could therefore have led to an underestimation of asymmetry compared to CONT. Despite this, we still found significant differences regarding asymmetry. It is therefore unlikely that gender differences would have influenced the main conclusions from our study.

### Squatting kinematics and kinetics

A significant difference in performance was shown by a reduced squat depth in both ACLD and ACLR compared to CONT (α_kn(mx)I_: ACLD and ACLR p < 0.001 and α_kn(mx)N_: ACLD p = 0.025 ACLR p = 0.035; Table 2). In ACLD peak knee extensor moments were significantly reduced compared to CONT in both the injured and the non-injured leg (*M*_*kn(mx)I*_: p = <0.001 and *M*_*kn(mx)N*_: p = 0.041), while in ACLR this was only significantly reduced in the injured leg (p < 0.001; Table 2). Despite the reduced squat depth and peak knee moments in ACLR and ACLD compared to CONT, their total support moment was not significantly reduced compared to CONT (*Msup*_*totI*_ and *Msup*_*totN*_; p = 0.109 and p = 0.152 respectively; Table 2). This suggests that in ACLR and ACLD the knee contributed to a lesser amount to the support moment compared to CONT. This was confirmed by our results as the percentage contribution of the knee to the total support moment was significantly reduced in the injured leg only in both ACLR and ACLD compared to CONT (*Msup*_*knI*_: ACLD and ACLR p < 0.001; *Msup*_*knN*_: ACLD p = 0.387, ACLR p = 0.324; Table 3; *Msup*_*knI*_: CONT: 44 ± 12; ACLR: 36 ± 10; ACLD: 36 ± 9). ACLD showed an increased contribution of the ankle to the support moment compared to CONT in both the injured and non-injured leg, and a decreased contribution of the hip in the non-injured leg (*Msup*_*ankI*_*, Msup*_*ankN*_ and *Msup*_*hipN*_; Table 3; p < 0.001). ACLR showed an increased contribution of the hip joint only in the injured leg (Table 3; p < 0.001). The reduced contribution of the knee was therefore mainly compensated for by an increased contribution of the hip. This was in agreement with findings by Salem et al. [14] in ACLR individuals. The reduction of the peak knee extensor moment in the injured compared to the non-injured leg was similar in ACLR in our study to that by Salem et al. [14] (we found a reduction of 20 % compared to a reduction of 32.6 % by Salem et al.). No studies have investigated this in ACLD individuals.

**Table 2:** Kinematics and kinetics

	***α***_***kn(mx)I ***_**(°)**	***α***_***kn(mx)N ***_**(°)**	***M***_***kn(mx)I ***_**(BW.h)**	***M***_***kn(mx)N ***_**(BW.h)**	***Msup***_***I ***_**(BW.h)**	***Msup***_***N ***_**(BW.h)**
CONT	113 ± 21	113 ± 21	0.059 ± 0.022	0.060 ± 0.022	0.143 ± 0.044	0.144 ± 0.042
ACLR	106 ± 17*	106 ± 17*	0.045 ± 0.015*	0.054 ± 0.014*	0.134 ± 0.042	0.140 ± 0.042
ACLD	105 ± 21*	106 ± 19*	0.046 ± 0.016*	0.061 ± 0.025	0.134 ± 0.036	0.150 ± 0.046

Mean peak knee flexion angles (*α*_*kn(mx)I*_ and *α*_*kn(mx)N*_), peak knee extensor moments (*M*_*kn(mx)I*_ and *M*_*kn(mx)N*_) and support moments at *M*_*kn(mx)*_ (*Msup*_*I*_ and *Msup*_*N*_) in the injured and non-injured leg respectively, with standard deviations for CONT, ACLR and ACLD. A * indicates a significant difference (p < 0.05) from CONT.

**Table 3:** Contributions of the ankle, knee and hip to the support moment

	***Msup***_***ankI ***_**(%)**	***Msup***_***ankN ***_**(%)**	***Msup***_***knI ***_**(%)**	***Msup***_***knN ***_**(%)**	***Msup***_***hipI ***_**(%)**	***Msup***_***hipN ***_**(%)**
CONT	20 ± 9	19 ± 9	44 ± 12	43 ± 11	38 ± 9	38 ± 9
ACLR	21 ± 11	19 ± 10	36 ± 10*	41 ± 11	43 ± 8*	40 ± 12
ACLD	27 ± 7*	25 ± 8*	36 ± 9*	41 ± 10	38 ± 9	34 ± 9*

Mean percentage contribution of the ankle, knee and hip joints to the total support moment with standard deviations. With *Msup*_*ankI*_*and Msup*_*ankN*_ the contribution of the ankle, *Msup*_*knI*_*and Msup*_*knN*_ the contribution of the knee and *Msup*_*hipI*_ and *Msup*_*hipN*_ the contribution of the hip in the injured and non-injured leg respectively. A * indicates a significant difference (p < 0.05) from CONT.

### Limb symmetry

These results indicate that even though the total support moments did not differ between the groups, there were differences between the injured and non-injured legs (asymmetries). To look further into these asymmetries, symmetry measures between the injured and non-injured leg were investigated. To define whether symmetry was near perfect, the decision rule was used that the rounded symmetry measure had to be 100. From our own data we have experienced that normal symmetry in healthy people fits within 5 % and our decision rule is meant to be consistent with this [31]. Symmetry of the peak knee flexion angle (*SYMα*_*kn(mx)*_) was close to 100 in all groups (CONT: 100 ± 3; ACLR: 100 ± 3; ACLD: 99 ± 3; Table 4) indicating near perfect symmetry between the injured and non-injured leg, as was expected in this closed chain exercise. *SYMα*_*kn(mx)*_ was not significantly different in ACLD and ACLR from CONT (ACLD: p = 0.057; ACLR: p = 1.000). *SYM*_*Msup*_ was significantly reduced in ACLD (p = 0.001), but not ACLR (p = 0.329), compared to CONT (Table 4). Symmetry of the support moment (*SYM*_*Msup*_) was close to 100 in CONT (99 ± 10); the support moment was therefore almost identical in both legs. In ACLD *SYM*_*Msup*_ was smaller than 100 (95 ± 12); the support moment was therefore reduced in the injured compared to the non-injured leg. *SYM%sup*_*kn*_ was significantly reduced in ACLD and ACLR compared to CONT (p < 0.001; Table 4). Symmetry of the % support moment produced by the knee (*SYM%sup*_*kn*_) was close to 100 in CONT (99 ± 9); the contribution of the knee to the total support moment was therefore almost identical in the injured and non-injured leg. *SYM%sup*_*kn*_ was lower than 100 for both ACLR and ACLD (93 ± 16 and 92 ± 13 respectively); therefore the knee contributed less to the support moment in the injured compared to the non-injured leg. *SYM%sup*_*ankle*_ was significantly reduced in ACLR (p = 0.030), but not ACLD (p = 0.242), compared to CONT (Table 4). Symmetry of the % support moment produced by the ankle (*SYM%sup*_*ankle*_) was 100 in CONT (100 ± 16); the contribution of the ankle to the total support moment was therefore identical in the injured and non-injured leg. *SYM%sup*_*ankle*_ was higher than 100 for both ACLR and ACLD (105 ± 18and 104 ± 14 respectively); therefore the ankle contributed more to the support moment in the injured compared to the non-injured leg. *SYM%sup*_*hip*_ was significantly reduced in ACLR (p = 0.005) and ACLD (p < 0.001), compared to CONT (Table 4). Symmetry of the % support moment produced by the hip (*SYM%sup*_*hip*_) was 100 in CONT (100 ± 11); the contribution of the hip to the total support moment was therefore identical in the injured and non-injured leg. *SYM%sup*_*hip*_ was higher than 100 for both ACLR and ACLD (105 ± 15and 106 ± 12 respectively); therefore the hip contributed more to the support moment in the injured compared to the non-injured leg. Overall this means that in CONT the total support moment was evenly distributed over the injured and non-injured leg and the ankle, knee and hip contributed to the total support moment in similar amounts in the injured and non-injured legs. In ACLR the total support moment was also similar distributed over the injured and non-injured leg, however the contribution of the knee was reduced and the contribution of the ankle and hip was increased in the injured compared to the non-injured leg. For ACLD the total support moment was reduced in the injured compared to the non-injured leg, also the contribution of the knee was reduced and the contribution of the hip was increased in the injured compared to the non-injured leg. This suggests the use of different motor control strategies in the different groups.

**Table 4:** Symmetry of kinematics and kinetics

	***SYMα***_***kn(mx) ***_**(%)**	***SYM***_***Msup ***_**(%)**	***SYM%sup***_***ankle ***_**(%)**	***SYM%sup***_***kn ***_**(%)**	***SYM%sup***_***hip ***_**(%)**
CONT	100 ± 3	99 ± 10	100 ± 16	99 ± 9	100 ± 11
ACLR	100 ± 3	97 ± 11	105 ± 18*	93 ± 16*	105 ± 15*
ACLD	99 ± 3	95 ± 12*	104 ± 14	92 ± 12*	106 ± 12*

Mean symmetry of the peak knee flexion angles between the injured and non-injured legs (*SYMα*_*kn(mx)*_), symmetry of the support moment between the injured and non-injured legs (*SYM*_*Msup*_), symmetry of the % support moment of the ankle (*SYM%sup*_*ankle*_), knee (*SYM%sup*_*kn*_) and hip (*SYM%sup*_*hip*_) between the injured and non-injured legs with standard deviations for CONT, ACLR and ACLD. A * indicates a significant difference (p < 0.05) from CONT.

### Squatting motor control strategies

**Figure 1:**
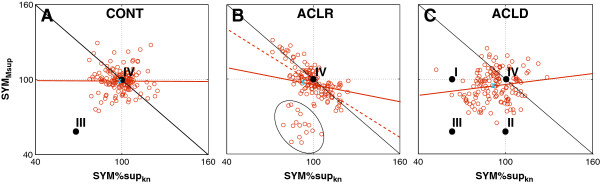
**Motor control strategies during double leg squat.** SYM_Msup_ versus SYM%sup_kn_ for: **A)** CONT, **B)** ACLR and **C)** ACLD. Combinations below the black solid line have a reduced knee moment in the injured limb. Points I-IV refer to the strategies identified in Figure 2, and the crosses refer to the average group data (Table 4). Adjusted R^2^: CONT = -0.007, ACLR all data: 0.098, ACLR without outliers (dotted line): 0.561, ACLD: 0.015. These outliers in ACLR were 2 subjects only.

The two main components that were varied in the different motor control strategies were *SYM*_*Msup*_ and *SYM%sup*_*kn*_. We therefore further investigated these motor control strategies in the different participant groups by looking at the relationship between *SYM*_*Msup*_ and *SYM%sup*_*kn*_ (Figure 1). There was no significant correlation between these variables in CONT (adjusted R^2^ = -0.007, p = 0.946; Figure 1A). Data were randomly distributed around a point close to IV (100,100; representing perfect symmetry in both legs, Figure 1). This variability would be expected in normal unconstrained performance. ACLR showed a small but significant correlation between *SYM*_*Msup*_ and *SYM%sup*_*kn*_ (adjusted R^2^ = 0.098, p < 0.001; Figure 1B). There were however two subjects that were behaving differently from the other ACLR subjects (circled data in Figure 1B). When these data were not included ACLR showed a significant and strong correlation between *SYM*_*Msup*_ and *SYM%sup*_*kn*_ (adjusted R^2^ = 0.561, p < 0.001; Figure 1B) and were distributed around a point close to IV (100,100). This was a negative correlation; therefore ACLR participants with the highest *SYM*_*Msup*_ presented the lowest *SYM%sup*_*kn*_ and vice versa. Whilst performing maximally, ACLR seemed constrained by the knee moment on the injured side. ACLR controlled the knee moment magnitude by using two strategies in combination; 1) transfer of support moment to the non-injured leg; 2) transfer of support moment from the knee to the ankle and hip of the injured leg. Different subjects combined these strategies in different proportions. The use of the strategies showed a negative correlation along the diagonal (dashed line in Figure 1B); therefore the increased use of one strategy would reduce the use of the other. The effect on the knee moment was therefore the same among subjects. ACLD showed no significant correlation between *SYM*_*Msup*_ and *SYM%sup*_*kn*_ (adjusted R^2^ = 0.015, p = 0.080; Figure 1C). The data were distributed around a point below 100 for both *SYM*_*Msup*_ and *SYM%sup*_*kn*_. ACLD therefore used an avoidance strategy where they reduced squat depth and subsequently the support moment in the injured leg and the contribution of the knee to this moment. The lack of correlation could be because some subjects were functioning better than others.

**Figure 2:**
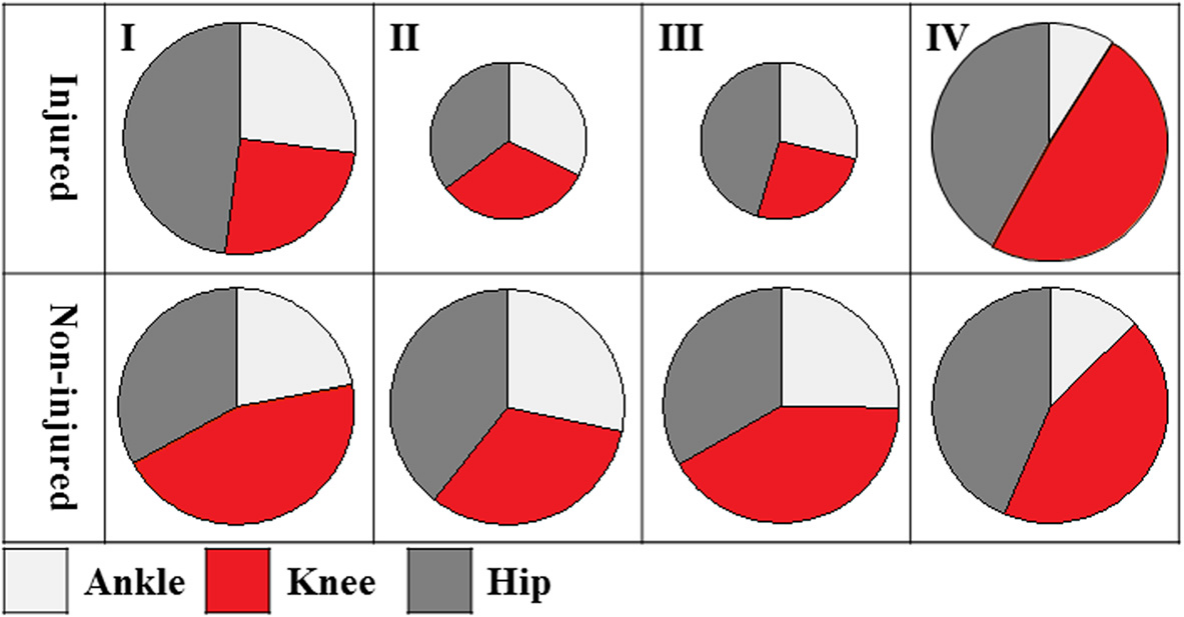
**Double leg squat compensation strategies (graphical representation).** The slices represent the percentage of support moment produced by the ankle (light grey), knee (red) and hip (dark grey). The sizes of the circles refer to the magnitude of the total support moment; the same size on the injured and non-injured side (I and IV) indicates an even distribution between the legs, while a smaller size circle on the injured side (II and III) indicates a reduced support moment in the injured leg. The strategies I-IV therefore represent the following: I) similar support moment in the injured and non-injured leg but reduced contribution of the knee in the injured leg (some ACLD individuals and ACLR), II) reduced support moment in the injured leg but similar contribution of the knee to the support moment in the injured and non-injured leg (some ACLD individuals and ACLR), III) reduced support moment and reduced contribution of the knee in the injured compared to the non-injured leg (some ACLD individuals only), IV) similar support moment and similar contribution of the knee in the injured and non-injured legs (some ACLD and some ACLR individuals and CONT).

From these results a general performance strategy of reducing depth of squatting and four different motor control strategies could be identified. These are graphically demonstrated in Figure 2: I) similar support moment in the injured and non-injured leg but reduced contribution of the knee in the injured leg (some ACLD individuals and ACLR), II) reduced support moment in the injured leg but similar contribution of the knee to the support moment in the injured and non-injured leg (some ACLD individuals and ACLR), III) reduced support moment and reduced contribution of the knee in the injured compared to the non-injured leg (some ACLD individuals only), IV) similar support moment and similar contribution of the knee in the injured and non-injured legs (some ACLD and some ACLR individuals and CONT).

## Conclusions

Despite having completed rehabilitation both ACLR and ACLD showed reduced double leg squat performance compared to CONT and used compensation strategies that would reduce loading of their injured knee. Different adaptation of motor control strategies were identified. ACLR demonstrated constrained behavior during a double leg squat to control knee moment magnitude. ACLD used an avoidance strategy with reduced performance, support moment and contribution of the knee to this moment in the injured leg. They both compensated with an increased contribution of the hip and ACLR also with an increased contribution of the ankle. The behavior in ACLD could potentially be explained by their reduced knee extensor and flexor strength and their increased fear of re-injury. The double leg squat is often used in early rehabilitation. This study demonstrated that ACLD and ACLR used different strategies compared to CONT. Attention needs to be paid as these patients may not exercise the injured leg as intended and squat depth on its own may not be adequate as a clinical outcome measure. By observation the exercise can appear normal, while the ACL injured individual may use kinetic compensation strategies. Therefore tools are needed to be able to use biomechanical information in assessment and treatment. These tools could involve manipulation of the support moment or ground reaction forces by means of kinematic adjustments which would be accessible to the clinical setting. The different strategies also highlight that individualized rehabilitation is essential. The long term implications of these findings are unknown. Clearly ACL injured individuals are not recovering movement strategies on early rehabilitation exercises. It could therefore be questioned whether rehabilitation should progress to more demanding exercising before this has been addressed and whether different types of exercises that directly address motor control should be included earlier in rehabilitation.

## Abbreviations

ACL: Anterior Cruciate Ligament; ACLD: Individuals with Anterior Cruciate Ligament deficiency; ACLR: Individuals with Anterior Cruciate Ligament reconstruction; CONT: Healthy control subjects; CSAS: Cincinnati Knee Rating System Sports Activity Scale; IKDC: International Knee Documentation Subjective Knee questionnaire; LASI: Left anterior superior iliac crests; LILC: Left lateral sides of the iliac crest; Mkn(mx): Peak knee extensor moments; Msup: Support moment at *M*_*kn(mx)*_; Msupank: The percentage of *Msup* produced by the ankle; Msupkn: The percentage of *Msup* produced by the knee; Msuphip: The percentage of *Msup* produced by the hip; RASI: Right anterior superior iliac crests; RILC: Right lateral sides of the iliac crest; SKnExt: Isokinetic knee extensor strength; SKnFlex: Isokinetic knee flexor strength; SYM%supankle: Symmetry of the % support moment of the ankle between the injured and non-injured legs; SYM%supkn: Symmetry of the % support moment of the knee between the injured and non-injured legs; SYM%suphip: Symmetry of the % support moment of the hip between the injured and non-injured legs; SYMMsup: Symmetry of the support moment between the injured and non-injured legs; SYMαkn(mx): Symmetry of the peak knee flexion angles between the injured and non-injured legs; TSK: Tampa Scale of Kinesiophobia; αkn(mx): Peak knee flexion angles.

## Competing interests

The authors declare that they have no competing interests.

## Authors’ contributions

All authors have made substantial contributions to the conception and design of this research and this manuscript and have provided substantial intellectual input. PR and KB have been involved in data acquisition and processing. KB has provided substantial support with subject recruitment. PR has performed all data analysis and prepared figures and tables for this paper. PR has drafted the manuscript. All authors have been involved in revising the manuscript critically and have given final approval of the version to be published.
